# Meeting at the Dreadnought Hospital

**Published:** 1918-03-09

**Authors:** 


### THE COLLEGE OF NURSING

**(Limited by Guarantee).**

### MEETING AT THE DREADNOUGHT HOSPITAL.

A MEETING to make better known the aims and objects of the College of Nursing was held at the Dreadnought Hospital, of the Seamen's Hospital Society, Greenwich, on Friday, February 22. Miss Gibson, a member of the Council of the College (formerly matron of the Poor-Law Infirmary, Birmingham), and Miss Cowlin, Assistant Secretary of the College (who attended in the place of Miss Rundle, the Secretary), addressed the meeting.

**Miss Gibson** said she would like first to state the points for which the College stood, and for which, in spite of much discussion and difficulty, the College had always stood, and intended to stand: (1) for State Registration of Nurses; (2) for a uniform curriculum; (3) for a one-portal examination. The war had brought under the notice of the nursing authorities the absolute chaos of the nursing profession. Every hospital, large and small, gave its own certificate and its own training, and every nurse went out into the world with a different idea of her profession and no absolute standard by which she could be passed. The variety in the training of nurses became more apparent to everyone when large numbers of nurses from all sorts of training-schools all over the kingdom were brought together and sent out to the war. That was probably the first thing which made the matrons of the large hospitals, and the persons who were selecting and sending out these nurses, realise the chaotic condition of the profession. A gathering of matrons and persons interested in nursing matters was held. The first conclusion that they came to was one that caused less friction than it would have done some years ago—the subject of State Registration. The matter was discussed, with the result that the College of Nursing was started.

### WHAT THE COLLEGE AIMS TO PROVIDE.

The College was out to provide methods for post-graduate instruction. A nurse who had finished her three years' training and had passed her one-portal examination, which gave her the opportunity of proving that she was fit for the work, would be helped to go on in her profession. Then it was hoped that the College would provide club-rooms, reading-rooms, and so on for the nurses. That, of course, was a side issue. It was hoped that it would provide recreation and companionship, opportunities for meeting together. Above all it was hoped that it would also provide a Benevolent Fund which would be sufficient to make nurses independent of their friends in the future. Nurses were very badly paid, and many nurses had friends to help. During this war the nation had incurred a great debt to nurses. They had given of their skill, their brains, their health, their tenderness. Miss Florence Nightingale founded the Nightingale Training-School, which was the first training-school really to give a definite and uniform training to nurses, as a tribute to the work of nurses during the Crimean War. It gave that training with the express proviso that the nurses were to be sent out into the world to pass on to others what they had received through the training-school. She (Miss Gibson) believed that the nursing profession of the future would be as much in advance of to-day as it was to-day in advance of the time when Miss Florence Nightingale founded her training-school.

### THE NEW COUNCIL.

The present Council of the College of Nursing was almost immediately undergoing dissolution. In May twelve of the members would retire; these vacancies would be filled by the votes of the nurses themselves, the members of the College. They would receive a ballot paper, and they would nominate anyone whom they wanted to sit upon the Council. The twelve persons to go on to the Council in May would be elected by the nurses themselves. She hoped all nurses who joined the College would realise that this was an enormous responsibility.

Next year another twelve members of the Council would retire, and the vacancies would be again filled by the members of the College, so at the end of two years after this next May the whole Council would have been elected by the nurses, and by the nurses alone. She thought this was an absolutely fair and democratic arrangement.

**Miss Cowlin** said the Seamen's Hospital was a splendid illustration of effort made towards the higher education of nurses. It had affiliated to it various hospitals for the treatment of women (the very word "affiliation" struck terror into the hearts of many organisations), and this could not be done without time and labour. The College of Nursing hoped in course of time to have a scheme of affiliation between the larger and smaller hospitals.

### A MEMBER'S RESPONSIBILITIES.

Application for membership had an immediate responsibility, because directly one joined a body one pledged oneself to fight its battles and to forward in every way its aims and objects. The College wanted every member to feel that she had a large organisation behind her which was going to back her up in any difficulty she might possibly meet with. A splendid programme was before the College.

**Mr. P. Michelli,** C.M.G. (secretary of the Seamen's Hospital Society), gave an interesting retrospect of nursing progress during the last thirty years, and concluded with an appeal to nurses to join the College: "It is better to belong to a great body than to fight for your own hand."

### WHERE NURSES FOUNDER.

There was something about nurses, continued Mr. Michelli, and especially women nurses, that always prompted them to get their ideas on to detail. They would always try to divert the issue by looking at some minor point. This was not the way in which this matter ought to be approached. It ought to be approached with the great idea that it was to be a profession of the nurses. Nurses should trust to others somewhat. They might trust to the women and to the members of the medical profession whose names they saw on that list, they should try not to carp at things they did not like, or because they did not approve of the way the money was raised, etc. The point was whether it was worth while joining a great profession and making oneself a member of it. That was the point to consider. He maintained that the College offered nurses a great opportunity. There was no desire to persuade. If the nurses came in they came in on their own responsibility. They should think it out carefully, and when they had come to the conclusion that it was the right thing to do, they should do it. The College provided a great chance, and they should seize it.

